# Increased dual-task interference during upper limb movements in stroke exceeding that found in aging – a systematic review and meta-analysis

**DOI:** 10.3389/fneur.2024.1375152

**Published:** 2024-07-05

**Authors:** Påvel G. Lindberg, Nadia AmirShemiraniha, Carmen Krewer, Marc A. Maier, Joachim Hermsdörfer

**Affiliations:** ^1^Institut de Psychiatrie et Neurosciences de Paris, INSERM U1266, Université Paris Cité, Paris, France; ^2^Department of Clinical Sciences, Karolinska Institutet, Danderyd University Hospital, Stockholm, Sweden; ^3^Chair of Human Movement Science, Department Health and Sport Sciences, School of Medicine and Health, Technical University of Munich, Munich, Germany; ^4^Department of Neurology, Research Group, Schoen Clinic Bad Aibling, Bad Aibling, Germany; ^5^INCC UMR 8002, CNRS, Université Paris Cité, Paris, France

**Keywords:** stroke, upper limb, ageing, dual-task, dual-task interference, cognitive-motor interference, hemiparesis, cognition

## Abstract

**Objective:**

To determine whether dual-task interference during upper limb tasks is increased in patients after stroke compared to healthy older subjects and to compare magnitude of stroke-induced change in interference to that explained by aging.

**Methods:**

We conducted a systematic literature search in MEDLINE, CINAHL, Google Scholar and PEDro databases up to October 2023 for studies on upper limb dual-tasks in stroke and elderly healthy subjects. Eleven upper limb dual-task studies in stroke patients and 11 studies in healthy older subjects were identified and systematically reviewed. A meta-analysis was performed on seven stroke studies and on five studies in healthy older subjects that included control groups.

**Results:**

Most stroke studies investigated proximal arm movements with kinematic measures, but few studies evaluated manual dexterity. In contrast, studies in healthy older subjects used more distal (finger tapping) tasks. The meta-analysis showed that stroke patients had on average a 19% (CI 95% = 1.0–37.3) increase in dual-task interference compared to age-matched healthy controls (*Z* = 2.06, *p* = 0.04). Older healthy subjects showed greater dual-task interference compared to younger subjects (19% greater, CI 95% = 6.5–31.2, *Z* = 2.98, *p* = 0.003).

**Conclusion:**

Meta-analysis revealed an increase in dual-task interference during upper limb movements in stroke patients, exceeding age-related changes, supporting the presence of subclinical impairments in divided attention post-stroke that may impede motor recovery.

## 1 Introduction

Upper limb sensorimotor impairments are common after stroke (1, 2), particularly impacting manual dexterity (3, 4). Impaired manual dexterity and finger control hampers essential grasping functions, impacting many everyday activities and autonomy (5–8). Approaches for measurement and targeted rehabilitation of dexterity are being developed, focusing on underlying impairments such as reduced finger force control, timing or independent finger movements [(9–11)]. A key aspect in these training approaches is the provision of enhanced hand and finger movement feedback, most often through enhanced visual feedback of movement performance. This increases attention to task performance, making the training more engaging (12, 13). However, attention to the task can be diminished through distraction. Visual distraction during visuomotor grip force control leads to less precise task execution in healthy subjects (14). In stroke, high distractibility is also associated with less precise grip force control (15). Performance in visuomotor tasks therefore also depends on selective (visual) attention, along with working memory and executive functions (16), which can be tested using dual-task paradigms.

Dual-task situations, i.e., concurrently performing a motor and a cognitive task, typically induce a performance decrement, even in healthy subjects, when compared to single task conditions (14, 17). This phenomenon has been termed dual-task interference and has also been demonstrated after stroke during walking (17, 18) and control of balance (19). Dual-task interference is supposed to result from limited cognitive resources (20, 21) that might affect (divided) attention, executive function, working memory and potentially other cognitive functions used during motor tasks. Many daily activities present dual-task situations (such as talking while dressing) and reduced capacity to perform dual-tasks can be detrimental for independence in daily life (22). Dual-task approaches in stroke may help elucidate the relative role and interaction of cognitive and motor dysfunction and inform on prediction of post-stroke motor recovery [(22–24)]. While dual-task interference during locomotion is well characterized in stroke patients (17, 22, 25), fewer studies have been dedicated to post-stroke dual-task interference during voluntary upper limb movements. Dexterous finger movements, being complex and involving high-level control, likely require greater cognitive resources than lower limb movements (26). Together, this suggests that upper limb dual-task interference may be greater than lower limb dual-task interference in stroke, although studies directly comparing the two are lacking. Enhancing the knowledge about cognitive mechanisms contributing to upper limb motor recovery after stroke is important to understand patients’ deficits in daily life, to devise stratification approaches for study design, for the development of prediction algorithms and for the development of targeted interventions. It is therefore central to synthesize the results on upper limb dual-task interference in stroke and to systematically review available studies and perform a quantitative meta-analysis.

To our knowledge, no systematic literature review has so far been undertaken on upper-limb dual-task interference in stroke patients. We had two aims: (1) to summarize the evidence for dual-task interference during upper limb movements in stroke patients (i.e., dual-task vs. single task) and to perform a meta-analysis on results comparing stroke patients with age-matched healthy controls, and (2) to assess dual-task interference also in healthy older persons, since most first-ever strokes occur in older persons, i.e., average age of first stroke 70y (27, 28) and to perform a meta-analysis on results comparing older versus younger healthy controls. Thus, age and stroke might be combining factors acting on dual-task interference. We asked four key questions: (i) Does dual-task interference occur in stroke patients using a dual-task paradigm including an upper limb task and a concurrent cognitive task? (ii) Is dual-task interference more marked in stroke patients compared to that in healthy age-matched control subjects? (iii) Do healthy older subjects show higher interference than healthy young subjects? And (iv) can task-or stroke-related variables be identified that influence the occurrence of dual-task interference?

We were particularly interested in studies that provide the degree (magnitude) of dual task interference for the group comparison since this permitted calculation of average degree of dual-task interference and statistical analysis through a meta-analysis. Therefore, this survey combines a systematical review with a meta-analysis in a sub-group of the selected studies.

## 2 Methods

We conducted a systematic review following the PRISMA reporting guidelines (29). We performed two literature searches using Pubmed, Cinahl, Google Scholar and Pedro databases for English studies up to 30 October 2023. The first search was dedicated to stroke patients, the second to healthy older subjects. The following keywords were used for the first search: ‘cognitive-motor interference AND stroke AND hand.’ For the second search the keywords were: ‘cognitive-motor interference AND older persons AND hand.’ We also performed separate searches with alternative keywords: ‘hand’ was replaced by ‘upper limb,’ ‘cognitive-motor interference’ was substituted by ‘dual-task,’ and ‘older persons’ was exchanged by ‘ageing’ or ‘aging.’ In PEDro we used cognitive-motor* stroke* upper limb* (or dual-task*, arm*). References in identified articles and reviews were also searched for relevant studies.

### 2.1 Selection of studies

**Figure 1:**
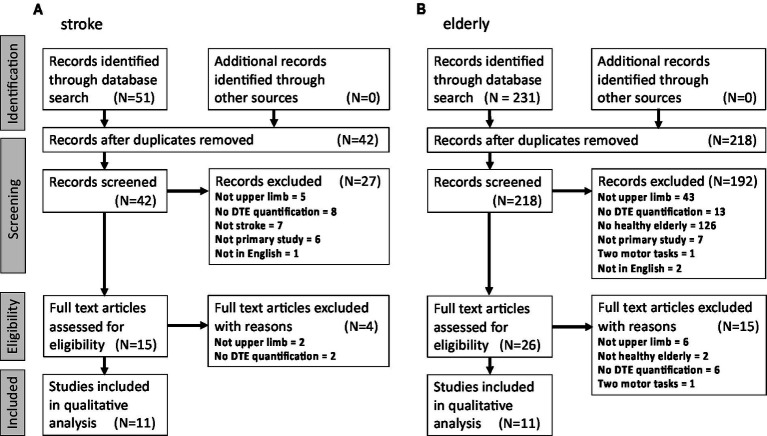
Flow diagram for the selection, inclusion and exclusion of upper limb studies on dual-task interference for the systematic review. **(A)** In stroke patients, and **(B)** in healthy older subjects. Screening stage exclusion criteria common to the two studies: Duplicates included article type that is not an original article (e.g., review, opinion etc.). Not in English: language other than English. Not upper limb: absence of upper limb motor task. No DTE quantification: absence of quantified dual-task effect. Two motor tasks: absence of a cognitive task in dual-task condition (i.e., two concurrent motor tasks). Specific to **(A)**: Not stroke: absence of stroke patients (i.e., studies on other or related disorders). Specific to **(B)**: No healthy older persons: absence of healthy older subjects (i.e., typically studies on older persons with clinical cognitive symptoms or other disorders). Not primary study: not an original article.

After deleting duplicates (from the alternative searches) the titles and abstracts were screened for eligibility (Figure 1). In the first search on stroke patients, articles were included if they met the following criteria: (1) assessment of dual-task ability while performing an upper limb and a cognitive task simultaneously, (2) study participants: adults with stroke, (3) presence of a measure of dual-task interference (an explicit quantitative measure or statistical test), (4) original research article written in English. In the second search, the same inclusion criteria were applied, except for the required study participants, which were healthy older subjects.

Exclusion criteria on publication type: reviews, meta-analyses, case reports, conference proceedings and abstracts, letters to the editor, and opinion papers. For the meta-analysis, studies without a control group or failing to report a quantitative measure of dual-task interference were excluded.

### 2.2 Data extraction for systematic review

Relevant data were extracted by authors (NA, CK, and MAM) and verified (PGL, JH). For the systematic review extracted data included information on study design, characteristics of patients and participants (e.g., sample size, gender, age, delay after stroke, baseline motor and cognitive function, Tables 1, 2), type of motor and cognitive tasks, outcome measures, and main qualitative dual-task interference results (Tables 3, 4). A quality assessment was undertaken according to the Critical Appraisal tools for use in JBI Systematic Reviews (52).

**Table 1:** Demographic characteristics of stroke population in selected studies.

Study	Group	*N*	Gender	Age (years)	Delay post-stroke	Motor status (severity)	Cognitive status	Study quality (JBI 0–9)
Bank et al. 2018 # (30)	Stroke	57	33 M / 24F	61 ± 10	Chronic (>24 m.)	Mild FMA-UE median = 57	Normal-to-mild MoCA median = 25	6
	Control	57	23F / 34 M	64 ± 8				
Bui et al. 2019 # (31)	Stroke (w/wo HIV)	3	1F / 2 M	55 ± 12	Chronic (>6 m.)	mild FMA-UE [52–64]	normal-to-Mild MoCA>24	5
	Control	5	3 M / 2F	[21–31]				
Dennis et al. 2011 (32)	Stroke	8	8 M	64 ± 12 [>43]	Chronic (>8 m.)	Mild-to-moderate FMA-UE > 41	n.a.	6
	Control	0						
Hejazi-Shirmard et al. 2020 # (33)	Stroke	34	18 M / 16F	57 ± 11	Chronic (mean = 37 m.)	Mild FMA-UE [50–61]	Normal MMSE mean = 27	7
	Control	34	18 M / 16F	57 ± 9				
Houwink et al. 2013 # (34)	Stroke	10	8 M / 2F	63 ± 8	Chronic (>2 m.)	Mild-to-moderate FMA-UE mean = 53 [32–66]	n.a.	7
	Control	10	8 M / 2F	62 ± 7				
Kim et al. 2021 # (35)	Stroke	10	0F / 10 M	55 ± 12	Chronic (> 5 m.)	Mild-to-moderate MMT mean = 66 FMA-UE mean = 41 [21–57]	Normal MMSE mean = 29 [26–30]	7
	Control	7	0F / 7 M	58 ± 11				
Lee et al. 2021 (36)	Stroke	13	3F / 10 M	46 ± 12	Chronic	n.a.	Normal-to-mild MMSE>24	6
	Control	0						
Mullick et al. 2021 # (37)	Stroke	13	6F / 7 M	64 ± 8	Chronic (> 7 m.)	Mild FMA-UE mean = 58 [51–64]	Normal-to-mild MoCA mean = 23 [18–28]	7
	Control	11	6F / 5 M	64 ± 11				
Pohl et al. 2011 (38)	Stroke	19		69 ± n.a. [>50]	Chronic (mean = 50 m.)	Mild FMA-UE mean = 58 ± 6	Normal MMSE mean = 28 ± 2	5
	Control	0						
Shin et al. 2017 (39)	Stroke	22	6F / 16 M	49 ± 12	Chronic (mean = 14 m.)	Mod.-to-severe FMA-UE mean = 29 ± 2	Normal MMSE mean = 28 ± 2	6
	Control	0						
Singh et al. 2023 # (40)	Stroke	16	4F / 12 M	62 ± 18	Chronic (> 6 m.)	n.a.	Mild impairment Visual Cognition Assessment mean = 15 ± 5	7
	Control	16	10F / 6 M	60 ± 10				

For motor and cognitive status: first line gives categorical status, second line gives respective clinical score (mean ± SD or median or threshold, range in []). MMSE, Mini-Mental State Examination; FMA-UE, Fugl-Meyer assessment – upper extremity; MMT, Manual Muscle Testing; MoCA, Montreal Cognitive Assessment; m., months; n.a., not available. All selected studies, except Lee et al. (36) and Shin et al. (39), had a cross-sectional design. #: study included in the meta-analysis. JBI quality appraisal: since 2 criteria (aimed at intervention studies) were not applicable to these studies, the range here is 0–7 (7 = max quality).

Search keywords: cognitive-motor interference/dual-task and stroke and hand/upper limb.

**Table 2:** Demographic characteristics of older persons in selected studies.

Study	Group	*N*	Gender	Age (years)	Motor status (severity)	Cognitive status	Study quality (JBI 0–9)
Corp et al. 2018 (41)	Old	15	9 M/6F	65 ± 4	n.a.	Normal-to-mild MMSE>24	7
	Control	15	9 M/6F	28 ± 3			
Ehsani et al. 2019 (42)	Old	79	n.a.	85 ± 5	FI: 23% non-frail	Normal-to-mild MoCA≥20	6
Kemper et al. 2003 # (43)	Old	75	n.a.	73 ± 6	n.a.	Normal-to-mild SPMSQ<4 errors	7
	Control	75		22 ± 2			
Mancioppi et al. 2021 (44)	Old	27	14 M/13F	65 ± 13	FI: 51% non-frail	Normal MMSE median = 29	6
Petit et al. 2011 # (45)	Old	15	n.a.	68 ± 8	n.a.	Normal MMSE>26	7
	Control	20	n.a.	23 ± 2			
Toosizadeh et al. 2016 (46)	Old	57	n.a.	81 ± 10	FI: 46% non-frail	Normal-to-mild MoCA≥20	6
Vaportzis et al. 2014 # (47)	Old	28	13 M/15F	72 ± 8	n.a.	Normal MoCa mean = 27.0 ± 1.9	7
	Control	28	13 M/15F	22 ± 3			
Voelcker-Rehage et al. 2006 # (48)	Old	14	8 M/6F	70 ± 4	Purdue Pegboard mean = 11.3 ± 3.0	Normal SILS mean = 35.3 ± 4.4, DSST mean = 54.6 ± 11.5, RDS-R mean = 7.6 ± 2.1	7
	Control	15	9 M/6F	20 ± 2			
Voelcker-Rehage et al. 2007 # (49)	Old	12	6 M / 6F	71 ± 2	Purdue Peg-board mean = 11.9 ± 2.2	Normal DSST mean = 58.9 ± 6.2, RDS-R mean = 7.9 ± 1.5	7
	Control	14	8 M/6F	22 ± 3			
Fujiyama et al. 2013 (50)	Old	12	6 M/6F	67 [60–75]	n.a.	Normal-to-mild MMSE≥24, mean = 28.8 ± 1.3	7
	Control	12	6 M/6F	21 [18–26]			
Heuninckx et al. 2004 (51)	Old	15	12 M/3F	64 [61–72]	n.a.	Normal MMSE≥26	7
	Control	15	6 M/9F	25 [22–31]			

For motor and cognitive status: first line gives categorical status, second line gives respective clinical score (mean ± SD or median or threshold, range in []). FI, Fried frailty index; SPMSQ, Short Portable Cognitive Status Questionnaire; SILS, Vocabulary subtest of the Shipley Institute of Living Scale; RDS-R, Reverse Digit Span subtest of the WAIS-R, others as in Table 1. Search keywords: cognitive-motor interference/dual-task and elderly/ageing/aging and hand/upper limb. All selected studies had a cross-sectional design. #: study included in the meta-analysis. JBI quality appraisal: since 2 criteria (aimed at intervention studies) were not applicable to these studies, the range here is 0–7 (7 = max quality).

**Table 3:** Single-and dual-task properties and performance outcomes in selected studies on stroke.

Study	Task			Dual task interference		Group differences (stroke vs. controls)			
	Upper limb	Cogn	Outcome measure	Controls (perf.)	Stroke (perf.)	Single task perf.		Dual-task perf.	
						Motor	Cognitive	Motor	Cognitive
Bank et al. 2018 (30)	Arm mvmt., frontal plane (proximal)	Auditory Stroop (percept. Discrim.)	Motor perf. (target count/mvmt. time); Cogn. %correct answers, reaction time	n.a. 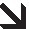	n.a.	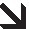 **	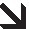 **	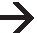	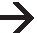
Bui et al. 2019 (31)	1 DoF arm tracking (robot, un-impaired side) (proximal)	Visual N-back (*N* = 4) (memory load)	Kinematics: *Smoothness of trajectory*, tracking error, velocity, acceleration, distance traveled, Cogn. count correct	n.a.	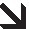 motor perf. ** 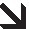 cogn perf. *	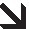 ***	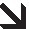 **	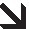 *n.a.	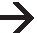
Dennis et al. 2011 (32)	Power grip force tracking (distal)	Clock-faces (percept. discrim.)	Kinematics: tracking error; Cogn. distinguish hands of the clock	No control group	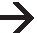 (but MCI trend)				
Hejazi-Shirmard et al. 2020 (33)	Reach-and-grasp (proximo-distal)	Backward digit task (memory load)	Kinematics: peak velocity, *mvmt. Time*, max. Grasp aperture; Cogn. incorrect number, order or omission error	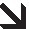 *	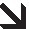 ***	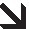 ***	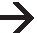	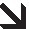 ***	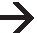
Houwink et al. 2013 (34)	Circle drawing (proximal)	Auditory Stroop (percept. Discrim.)	Kinematics: mvmt. Speed, mvmt. Accuracy (speed*accuracy)	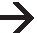	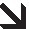 motor perf. (mod. Affected only) *n.a.	n.a.	n.a.	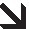 *	n.a.
Kim et al. 2021 (35)	planar robot (horiz.) arm mvmt. (proximal)	Digit span test and COWAT (memory load)	Kinematics: mvmt. Speed, *mvmt. Accuracy*; Cogn. correct answers, *speed*	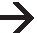 motor perf. 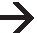 cogn perf.	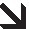 motor perf. * 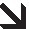 cogn perf. *	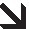 *	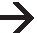	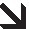 *	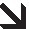 *
Lee et al. 2021 (36)	planar robot (horiz.) arm mvmt. (proximal)	Digit span test and COWAT (memory load)	Kinematics: smoothness of trajectory, path error, mvmt. Velocity, reach error	No control group	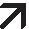 motor perf. With training *** 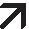 cogn perf. With training ***				
Mullik et al. (2021) (37)	3D-reaching in VR (proximal)	Auditory-verbal 1-back (memory load)	Motor perf: success rate; Cogn perf: success rate	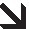 motor perf. *** 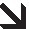 cogn perf. ***	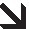 motor perf. *** 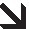 cogn perf. ***	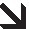 *	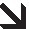 *	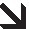 *	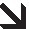 *
Pohl et al. 2011 (38)	Thumb clicking (distal)	Speaking / walking (language)	Motor perf: click rate; Cogn perf: word count	No control group	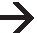				
Shin et al. 2017 (39)	Planar reaching (robot) (proximal)	Digit span test or COWAT (memory load)	Kinematics: *smoothness*, velocity path error, reach error	No control group	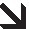 in severely impaired *				
Singh et al. 2023 (40)	Planar reaching (robot) (proximal)	Alpha-numeric switching (Trail-making test)	Kinematics: reach speed	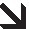 motor perf. *n.a.	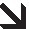 motor perf. *n.a. 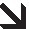 cogn perf. *n.a.	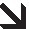 *n.a.	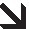 *	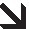 *	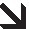 *

Dual-task interference: statistically significant changes in performance (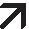 increased, 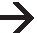 similar, 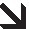 decreased) is indicated with respect to single-task performance (of the same group, i.e., within Stroke patients, and within Controls). *, **, *** indicate *p*-value of < 0.05, <0.01, and < 0.001, respectively. *n.a. indicates *p*-value not available (but difference stated as being significantly different in cited reference). Statistically significant group differences: single-task and dual-task performance (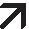 increased, 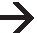 similar, 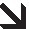 decreased) of Stroke patients with respect to Controls, separately for cognitive and motor performance. Motor tasks were performed with the affected limb (otherwise indicated). If there are several outcome measures in a given motor or cognitive task: variables in italics are those used for comparison. perf., performance; mvmt., movement; coord., coordination; VR, virtual reality.

**Table 4:** Single-and dual-task properties and performance outcomes in selected studies on older persons.

Study	Task			Dual task interference		Group differences (Older vs. Controls)			
	Upper limb	Cogn	Outcome measure	Controls (perf.)	Older (perf.)	Single task perf.		Dual-task perf.	
						Motor	Cognitive	Motor	Cognitive
Corp et al. 2018 (41)	Visuo-motor arm tracking (proximal)	n-back, verbal fluency, (memory load)	Kinematics: elbow angle; Cogn: %correct responses	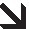 cogn perf. *	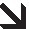 motor perf. * 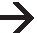 cogn perf.	n.a.	n.a.	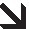 *	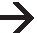
Ehsani et al. 2019* (42)	Cyclic elbow flexion (proximal)	Backward counting by 1 or 3 (memory load)	Kinematics: speed, ROM, *speed variability*, ROM variability	No young control group	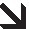 perf. (trend)				
Kemper et al. 2003 (43)	Finger tapping task (distal)	Talking: respond to questions (language)	Motor perf. (*tapping rate*, sequence errors); Cogn perf. (speech onset, speech fluency and complexity)	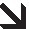 motor perf. * 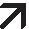 cogn perf. **	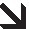 motor perf. * 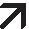 cogn perf. **	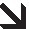 *	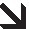 **	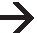	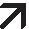 **
Mancioppi et al. 2021 * (44)	Index finger tapping at own pace (distal)	Backward counting by 1, 3 or 7 (memory load)	Kinematics: *opening velocity*; Kinetics: jerk Perf: tapping count	No young control group	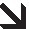 motor perf. *n.a.				
Petit et al. 2011 (45)	Alternating index / middle finger tapping (distal)	Letter fluency (language)	Motor perf: tapping reaction time; Cogn: word count	MCI trend	MCI trend	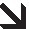 ***	n.a.	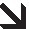 ***	n.a.
Toosizadeh et al. 2016 * (46)	Cyclic elbow flexion (proximal)	Backward counting by 1 (memory load)	Kinematics: ROM, speed, *speed variability*; Cogn: correct count	No young control group	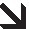 motor perf. (trend)				
Vaportzis et al. 2014 (47)	Upper limb circle tracing (tracking) (proximal)	Serial subtraction (by 2: easy; or 3: hard) (memory load)	Kinematics: *drawing speed*, drawing accuracy Cogn: error rates, response rates	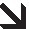 motor perf. *n.a. (but 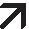 motor perf. For accuracy)	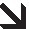 motor perf. *n.a. (but 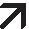 motor perf. For accuracy)	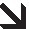 *n.a.	n.a.	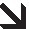 *	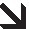 **
Voelcker-Rehage et al. 2006 (48)	Steady-state precision grip force (distal)	n-back (memory load)	Kinetics: variability of force maintenance; Cogn: % correct responses	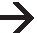 motor perf. 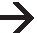 cogn perf.	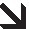 motor perf. * 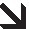 cogn perf. *	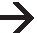	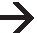	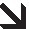 *	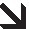 **
Voelcker-Rehage et al. 2007 (49)	Precision grip sine-wave force tracking (distal)	n-back (memory load)	Kinetics: variability of force modulation; Cogn: correct responses	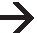 motor perf. 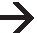 cogn perf.	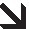 motor perf. ** 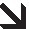 cogn perf. **	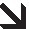 **	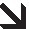 **	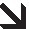 **	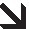 *
Fujiyama et al. 2013 (50)	Cyclical elbow and knee flex/ext. (proxim. Interlimb coord.)	Auditory-verbal reaction time task	Kinematics: amplitude, frequency, *accuracy;* Cogn: verbal reaction time	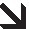 cogn perf. ***	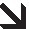 cogn perf. ***	n.a.	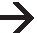	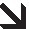 **	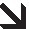 **
Heuninckx et al. 2004 (51)	Cyclical wrist and ankle flex/ext. (distal interlimb coord.)	Memorize visual target figure (memory load)	Kinematics: relative phase, peak-to-peak amplitude, *max coordinated flex/ext frequency*; Cogn: error count	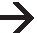 motor perf.	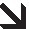 motor perf. *	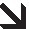 *n.a.	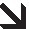 *n.a.	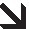 *n.a.	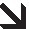 *n.a.

Dual-task interference: statistically significant changes in performance (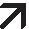 increased, 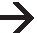 similar, 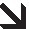 decreased) is indicated with respect to single-task performance (of the same group, i.e., within older subjects, and within younger Controls). Statistically significant group differences: single-task and dual-task performance (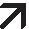 increased, 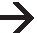 similar, 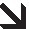 decreased) of older subjects with respect to Controls, separately for cognitive and motor performance. *, **, *** indicate *p*-value of < 0.05, <0.01, and < 0.001, respectively. *n.a. indicates p-value not available (but difference stated as being significantly different in cited reference). If there are several outcome measures in a given motor or cognitive task: variables in italics are those used for comparison.

perf., performance; mvmt., movement; coord., coordination; flex/ext, flexion/extension. * study without a young control group.

### 2.3 Meta-analysis

Meta-analysis was performed on a sub-population of the selected studies for the systematic review (Tables 1, 2). Additional inclusion criteria for meta-analysis: presence of a control group, and of a quantified measure of dual-task interference applied to the test and the control group. Data extraction for meta-analysis included: number of healthy subjects/stroke patients and amount of mean dual-task interference (and SD) for each group and study. Dual-task interference was normalized to single-task performance and expressed in (%) as follows:

((Motor_performanceSingleTask – Motor_performanceDualTask) / Motor_performanceSingleTask)*100

In case of missing mean dual-task interference values, they were calculated from other available data (e.g., Figures) or after having received the data by contacting the corresponding author. In two studies, data only presented in figures were extracted by means of WebPlotDigitizer, v4.6.^1^ Whenever more than one motor task, several cognitive tasks or more than one motor performance parameter were investigated, the task/parameter showing the greatest dual-task interference was used for the meta-analysis. Supplementary Tables 1, 2 provide detailed information on the specific data used for the meta-analyses.

Data were analyzed using Review Manager (RevMan)5.4.1. Forest plots were used to represent the results of the meta-analysis. Given the clinical heterogeneity of the selected studies, and their often small sample size, random-effects models [not requiring the assumption of normality for the random effect; (53)] were used to calculate the pooled estimate of the dual-task interference and the associated 95% confidence interval (CI). Statistical heterogeneity across the studies was evaluated using *I*^2^ (>75% considerable heterogeneity). For the mean difference approach, the SDs are used together with the sample sizes to compute the weight given to each study. Studies with small SDs are given relatively higher weight whilst studies with larger SDs are given relatively smaller weights, and in a heterogeneous set of studies, a random-effects meta-analysis will award relatively more weight to smaller studies.

## 3 Results

### 3.1 Study selection – on stroke patients

The initial literature search found 51 references for stroke patients. After removal of duplicates 42 remained. Screening, based on Title and Abstract, resulted in the exclusion of 27 articles. All of the remaining 15 articles were full text screened and 11 retained for systematic review (Figure 1A). Meta-analysis was performed on 7 of the articles (4 studies included in the systematic review did not report or did not include a healthy control group, Table 1).

### 3.2 Stroke – description of studies

Ten of the included studies were cross-sectional and one was longitudinal (Table 1). A control group of healthy subjects was present in 7 out of 11 studies. A total of 205 stroke patients and 140 age-matched healthy control subjects were included across the 11 studies. Studies with groups showing a difference in mean age < 10 years were considered age-matched. The median sample size of included stroke patients was *N* = 13 (range: *N* = 3–57). Characteristics of stroke subjects: most studies included more male than female subjects, mean age of patients varied from 46 to 69 years, all patients were included in the chronic phase (most studies >6 months, 1 study >2 months post-stroke; Table 1). Motor status (upper limb severity) was mostly assessed with the FMA-UE and sensorimotor impairments mostly varied from mild-to-moderate. Only one study included severely affected patients. Two studies did not report motor impairment. Cognitive status was usually assessed with either the MMSE or MOCA, and stroke patients had either intact cognitive functioning or had mild impairment.

### 3.3 Stroke – tasks and outcome measures

The upper limb motor tasks and performance measures of the 11 studies are shown in Table 3. Eight studies used a *proximal* motor task requiring shoulder/elbow control, e.g., free arm movement in the frontal plane, arm tracking using a robot, planar horizontal reaching and circle drawing. One study used a mixed *proximo-distal* task with reach-and-grasp or tool handling, and two studies used purely *distal* tasks, such as power grip force tracking or thumb clicking. None of the studies used a distal task requiring manual dexterity. In terms of task design, ten studies used visuo-motor tasks and one study used an audio-motor task. Outcome measures also varied considerably across studies, with 7 studies reporting kinematic or kinetic measures and 4 studies (40%) using more global performance measures. Eight studies quantified performance with movement speed measures (including velocity and reaction time) and 7 studies reported movement precision measures (including precision and tracking error).

Cognitive tasks also varied widely across the 11 studies (Table 3). Under dual-task conditions, the various cognitive tasks generally increased the demand on executive function and on selective attention. However, each cognitive task also involved specific cognitive functions. Thus, perceptual discrimination was engaged by tasks such as Stroop (two studies) and audio-visual clock-faces (one study); in contrast, memory load was increased by tasks such as N-back (one study) and mental digit or alphanumeric sequences (three studies). Finally, some studies used language tasks involving speaking (one study) or word associations (three studies).

Regarding dual-task instructions, none of the studies provided explicit instructions to prioritize one task over the other (motor vs. cognitive).

### 3.4 Stroke – dual-task interference

Dual-task interference, i.e., reduced performance during dual-task, was present in stroke patients in 10 studies (Table 3). Only the study by Pohl et al. (38) did not find a dual-task interference in stroke patients. Of these 10 studies, 7 included control subjects: and the control group showed a dual-task interference in 4 out of 7 studies. Thus, not all studies showed consistent dual-task interference in stroke patients and in controls. An increased dual-task interference (in qualitative or statistical terms) was present in stroke patients, relative to controls, in 5 out of 7 studies.

### 3.5 Stroke – meta-analysis

**Figure 2:**
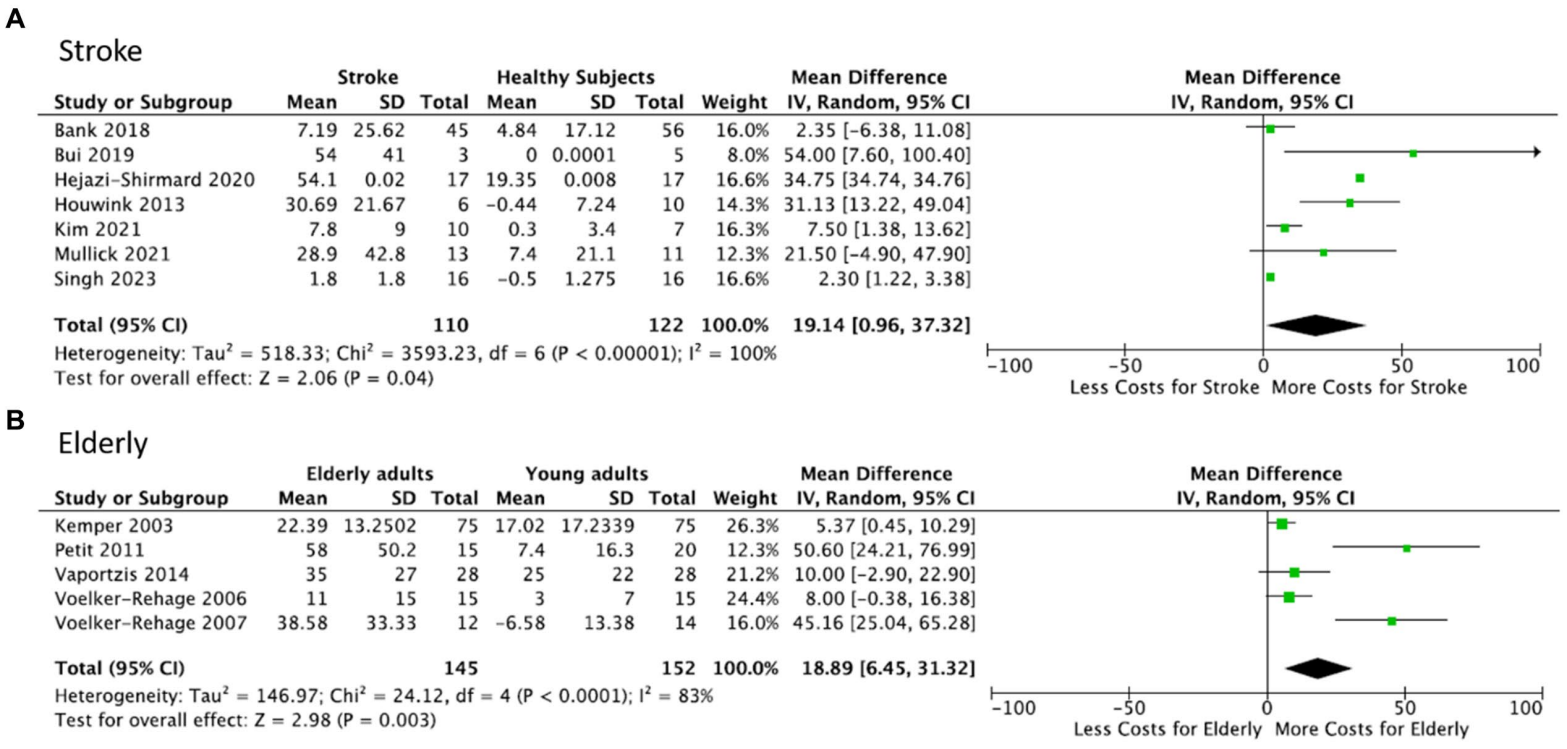
Meta-analysis: Forest plot for dual-task interference in upper limb tasks (group comparison). **(A)** Stroke patients vs. healthy controls. Overall dual-task interference was significantly increased (indicated by “greater cost” during performance of motor task) instroke patients relative to age-matched controls. **(B)** Healthy older subjects vs. healthy younger subjects. Overall dual-task interference, indicated by a positive dual-task cost, is significantly stronger in healthy older subjects relative to young healthy controls. Column headings in the two Tables: Mean dual-task interference in stroke patients **(A)** and older subjects **(B)** is expressed as the difference in dual task interferences. SD: standard deviation of Mean. Total: number of subjects.

The meta-analysis (Figure 2A) showed significantly higher overall dual-task interference in the stroke group compared to the age-matched healthy controls (7 studies, mean dual-task interference difference = 19.14, 95% CI = 0.96–37.32, *p* = 0.04). The heterogeneity was high (*I*^2^ = 100%). Detailed description of task parameters and performance variables to quantify interference are given in Supplementary Table 1.

### 3.6 Study selection – on older persons

The literature search for the older persons yielded 231 references, 13 of them being duplicates. After screening the Titles and Abstracts, 26 articles were selected for full-text screening and 11 retained for systematic review (Figure 1B). A meta-analysis was performed with 5 of the studies (6 studies were excluded: 3 studies had no younger control group, 2 studies reported inter-limb coordination tasks rather than unilateral upper limb tasks, and Corp et al. (41) did not provide sufficient data to calculate dual task interference, Table 3).

### 3.7 Older persons – description of studies

All 11 included studies had cross-sectional designs (Table 2). Two types of control groups were used: younger people (8 studies) and older people with cognitive impairments (3 studies). Results in older subjects with cognitive impairments are not reported in this review. In summary, 349 healthy older people and 194 young people were enrolled within the 11 studies. The median sample size of the included older subjects was *N* = 32 (range: *N* = 12–79). Characteristics for healthy older subjects: most studies included more males than females and mean age of older subjects varied between 65 and 85 years. Clinical motor function was typically measured using the Purdue Pegboard Test. Manifestation of frailty was assessed using the Fried Index. Studies reported on subjects with no-to-mild age-related frailty and impairments in manual dexterity. Cognitive function was assessed using the MMSE or the MoCA, and for the included older persons, studies reported normal (or mildly impaired) cognitive function.

### 3.8 Older persons – tasks and outcome measures

Most upper limbs tasks used in the 11 included studies were *distal* tasks (Table 4), such as force-tracking (2 studies) and finger tapping (3 studies). Cyclic inter-limb coordination tasks (e.g., flexion-extension of wrist and ankle) were used in two studies (2 studies). The remaining four studies employed *proximal* effector tasks including cyclic elbow flexion (2 studies), upper limb circle tracing (1 study) and arm movement tracking (1 study). Moreover, 5 out of 11 studies used visuo-motor tasks and one study used an audio-motor task. All 11 studies used kinematic outcome measures. Seven studies quantified performance with movement speed measures (including movement frequency, velocity and reaction time), 5 studies reported movement amplitude measures (including range of movement, and angle measures), and 3 studies reported movement variability measures.

Cognitive tasks included working memory tasks such as counting backwards (3 studies), serial subtraction (1 study) and versions of the n-back (3 studies) task. One task (1 study) requested memorizing visual shapes (1 study). Language tasks included letter fluency (1 study), answering questions orally (1 study), or providing verbal response to auditory stimuli (1 study).

Overall, most studies (9 studies) did not provide explicit information on which task to prioritize (motor vs. cognitive). But two studies did: in Heuninckx et al. (51) participants were advised to prioritize the attentional task, whereas in Fujiyama et al. (50) the motor task was declared the primary task.

### 3.9 Older persons – dual-task interference

All 11 studies reported a dual-task interference in older participants, at least qualitatively (Table 4). Of the 8 studies with a young control group, 6 studies reported a dual-task interference in younger healthy subjects. In the majority of studies with young control groups (7 out of 8 studies), the dual-task interference was more pronounced in older compared to that in younger subjects (i.e., decreased motor performance in the group difference, Table 4).

### 3.10 Older persons – meta-analysis

The meta-analysis (Figure 2B) showed significantly higher overall dual-task interference in older healthy subjects compared to the younger healthy subjects (5 studies, mean dual-task interference difference = 18.89, 95% CI = 6.45–31.32, *p* = 0.003). Heterogeneity was high (*I*^2^ = 83%). Data and variables used in the meta-analysis for healthy older subjects are presented in Supplementary Table 2.

## 4 Discussion

We performed a first meta-analysis on dual-task interference in stroke patients compared to age-matched healthy subjects when performing upper limb movements and concomitant cognitive tasks. The results showed a significantly increased dual-task interference in stroke patients with a 19% average increase in dual-task interference compared to age-matched healthy subjects. The systematic review provides a first description of upper limb dual-task studies in stroke and aging, providing details on study design and tasks used and on qualitative outcome. For example, stroke studies mostly used proximal arm movements with kinematic measures whereas studies on healthy older subjects used more distal (e.g., finger tapping) tasks. Cognitive context of upper limb tasks is therefore important to consider and cognitive-motor interactions may represent an important mechanism of upper limb motor recovery post-stroke and could be useful for the development of prediction algorithms and personalized interventions.

### 4.1 Increased dual-task interference in upper limb tasks in stroke

The meta-analysis results were partly expected given the greater occurrence of dual-task interference found in the systematic review (Table 3). These findings are in line with reports of increased dual-task interference in lower limb tasks such as gait and balance after stroke (25). Our study also shows that the stroke related increase in dual-task interference goes beyond that explained by age-related decline in dual-tasking. For the large majority of studies [the study of Mullick et al. (37) is an exception reporting a mean MoCA score of 23, below the cut-off for normal cognition of 26], the impaired cognitive-motor interactions occurred in stroke patients without cognitive impairments according to clinical assessments (MoCA and MMSE ranges in patients, see Table 1), suggesting presence of subclinical deficits in divided attention and executive function. It is also likely that more elaborate cognitive tests would have revealed attentional deficits in these patients. These findings agree with previous studies on visual distraction showing that reduced visuospatial attentional processing affects manual visuomotor task performance in stroke (15), and that cognitive functions are important for upper limb motor recovery after stroke (54), especially for more complex distal hand movements (24).

In terms of potential neural correlates, in healthy subjects, dual-tasking involves motor areas, prefrontal and parietal cortex and cerebellum (56). In stroke patients, one of the reviewed studies using functional MRI reported positive correlations between contralesional premotor (and prefrontal) cortex activity and degree of dual-task interference (32). Similarly, during dual-task locomotion, stroke patients showed increased prefrontal cortex activity, assessed by near-infrared spectroscopy (NIRS), compared to single-task locomotion (55). Thus, stroke lesions to the parieto-frontal circuitry, essential for cognitive-motor actions and executive control (57), likely contribute to increased dual-task interference post-stroke. Furthermore, stroke damage to this cortical network may also evoke subclinical attentional deficits (58). Another possibility is white matter damage to networks involved in sensorimotor integration: the longitudinal superior fasciculus has been shown to be important for visuomotor integration during manual tasks (59, 60), and larger white matter lesions affecting sub-cortical structures (basal ganglia) have been shown to be detrimental to post-stroke dual-task gait performance (61, 62). However, no studies have yet related lesion location to upper limb dual-task performance in stroke.

Since many activities of daily living are performed in dual-task situations (e.g., talking while dressing, or listening to the radio while preparing a meal) reduced upper limb motor performance in dual-tasks could be detrimental for independence and quality of life. Our findings thus support the need to measure dual-task capacity post-stroke. Dual-tasks may also offer novel avenues for training (63), although no upper limb dual-task training studies in stroke have yet been undertaken.

### 4.2 Role of task properties for detection of dual-task interference

It is plausible that the degree of interference depends on the characteristics of both the motor and of the cognitive task, and of their interaction. Upper limb motor tasks varied widely, (i) in terms of outcome measure (with potentially different sensitivity to changes in movement performance), (ii) in terms of task difficulty (i.e., increasing difficulty requires more attentional resources), and (iii) in terms of movement effector (proximal vs. distal upper limb).

First, considering outcome measures, studies that used kinematic (or kinetic) measures, rather than overall performance (e.g., number of correct trials), tended to show the strongest dual-task interference. This was the case in stroke (vs controls, Figure 2A), such as in Bui et al. (31), Hejazi-Shirmand et al. (33) or Houwink et al. (34), who all measured movement velocity (or smoothness). This is mirrored on a qualitative level in Table 3, where all studies using kinematic measures showed decreased dual-task motor performance in stroke patients [except for one which, however, used a training protocol, (36)]. In studies on older persons (vs. young), the efficacy of kinematic variables was less clear in the analysis. Here the strongest interference difference (Figure 2B) was found for a non-kinematic performance measure [reaction time, (41)], but the second strongest used a kinetic measure (49). Qualitatively, Table 4 indicates that older persons showed decreased dual-task performance in all studies using kinematic/kinetic measures, but the few studies using outcome measures also showed such a decrease.

Second, motor task difficulty is a further parameter to consider, as suggested by dual-task locomotion studies showing strongest interference during the most difficult locomotor tasks, e.g., in obstacle avoidance (25). In stroke (Figure 2A), a study requiring movement tracking (31) showed strongest interference (compared to controls). Weaker differences in interference (between patients and controls) was present in reaching (33) or circle drawing (34). A similar pattern was found in the studies on older persons: tasks requiring visuomotor tracking, i.e., continuous attention, tended to evoke larger differences in interference between groups. For example, Voelcker-Rehage et al. (49) used precision grip force tracking and reported substantial interference, whereas Vaportzis et al. (47) reported less interference in an arm circle drawing (tracking) task, where a speed-accuracy trade-off (63) may have interfered. A strong dual-task effect was also found during alternating index-middle finger tapping at maximal speed (41), a task that involves sustained attention and is sensitive to age-related decline (64). In contrast, weaker difference in interference was present in sequential finger movements, i.e., memorized movement sequences (43) and in steady-state (i.e., predictable) grip force maintenance (48). Although motor task difficulty, measured using speed-accuracy tradeoff during reaching, may partially explain stroke related reaching deficits under single-task conditions (65), it is less clear how motor and cognitive task difficulty mutually interact (parametrically) under dual-task conditions.

Third, movement effector is another parameter to be taken into account. We distinguished (in a simplifying approximation) distal from proximal upper limb tasks (Tables 3, 4). In the stroke studies, proximal tasks tended to generate stronger interference (compared to control, Figure 2A), as in Bui et al. (31) and in Houwink et al. (34), or proximo-distal as in Hejazi-Shirmard et al. (33). In contrast, in older persons, distal tasks, i.e., precision grip force control (49) and alternating index-middle finger tapping (41) evoked stronger interference (Figure 2B). The difference between stroke patients (proximal) and older persons (distal) is likely related to stroke typically affecting the distal upper limb (1).

Taken together, difficult motor tasks, i.e., those requiring continuous attention, typically for on-line tracking and correction, evoked stronger differences in dual-task interference between stroke patients and healthy controls. Kinematic/kinetic outcome measures seem to be more sensitive for detecting interference than global performance measures. And proximal upper limb tasks in stroke, but distal tasks in older persons seem to favor interference.

In addition, the type and difficulty of the concomitant cognitive task also plays a role in producing dual-task interference. A wide variety of cognitive tasks were used in the selected studies, but n-back or serial subtraction tasks engaging the working memory were used most frequently, both in studies on stroke and on older persons. No clear pattern between type of cognitive task and degree of dual-task interference was apparent, neither for stroke (Table 3), nor in older persons (Table 4), nor for strongest interference difference against control subjects (Figure 2).

### 4.3 Motor and cognitive impairment level contributing to dual-task interference

The degree of dual-task interference may directly relate to motor and cognitive symptom severity post-stroke. However, Houwink et al. (34) compared dual-task interference on motor task performance in patients with mild vs. moderate FMA-UE motor symptoms and found that only those with moderate symptoms had an increased dual-task interference relative to that of control subjects, but not patients with mild symptoms. Most of the studies included patients without clinical cognitive symptoms (Table 1). Five studies explicitly attempted to relate motor-and/or cognitive symptom severity to frequency and/or strength of dual-task interference: four studies found no relation between dual-task interference and motor impairment (FMA-UE score) (32, 33, 37, 39), and one (30) observed a positive correlation between dual-task interference and a combined motor-cognitive severity score. On the cognitive side, one study (33) indicated that high anxiety stroke patients showed stronger dual-task interference than low anxiety patients.

### 4.4 Limitations

Our systematic review on stroke subjects identified only 11 studies, with 7 of them included in the meta-analysis. This may impact the generalizability of the results. The studies included chronic patients with varying, typically mild-to-moderate post-stroke motor impairments, and normal-to-mild cognitive status. Future dual-tasking studies in the sub-acute post-stroke phase and among patients with severe motor impairment are indicated to explore whether time post-stroke and motor impairment severity affect cognitive-motor performance.

Another limitation is the small sample size across studies (median *N* = 13 patients, min = 3), which reduces the statistical power of the analysis. However, the meta-analysis weighs the results according to sample size of each included study and thus avoids disproportional contribution of small samples to the reported total interference effect (Figure 2A). Similar concerns need be considered for the review on older persons: few (11) studies were identified, although sample size was larger (median *N* = 32 older persons).

The heterogeneity of motor and cognitive tasks used to test dual-tasking throughout these studies may have introduced bias to the findings. For instance, studies in older healthy subjects tended to use more dexterous tasks, whereas arm reaching with robots was more common in stroke studies. Different performance measures were employed across studies for assessing dual-task performance (Tables 3, 4). These methodological variations likely account for the substantial heterogeneity found in the meta-analysis, underscoring the need for caution when interpreting the relevance and generality of these findings. Finally, another patient heterogeneity factor that could have influenced results was lesion location: lesions to parieto-frontal networks may be associated with greater dual-task interference (32, 55, 56).

## 5 Conclusion

The systematic review and meta-analysis show that persons with stroke, even in the absence of clinical signs of cognitive impairment, exhibit heightened susceptibility to dual-task interference when using their (affected) upper limb concurrently with a cognitive task. This heightened interference likely results from lesions to the parieto-frontal circuitry. Healthy older persons, about the age of first-ever stroke, showed stronger dual-task interference compared to young control subjects. This suggests that decreased dual-task performance in stroke subjects is due to the combined effect of stroke and aging. Generally, the nature and difficulty of the motor task seem to influence the degree of decrement in dual-task performance. More complex motor tasks, particularly those requiring sustained attention for on-line movement control, tend to produce the strongest dual-task interference. However, these findings should be interpreted cautiously given the small sample size and heterogeneity of experimental approaches in the selected studies. Open issues to be addressed concern dual-task interference in manual dexterity post-stroke and understanding the relationship between dual-task interference and upper limb motor recovery after stroke.

## Data availability statement

The original contributions presented in the study are included in the article/Supplementary material, further inquiries can be directed to the corresponding author.

## Author contributions

PGL: Conceptualization, Funding acquisition, Investigation, Methodology, Supervision, Writing – original draft, Writing – review & editing. NAS: Data curation, Investigation, Validation, Writing – original draft, Writing – review & editing. CK: Formal analysis, Investigation, Methodology, Software, Validation, Writing – review & editing, Writing – original draft. MAM: Conceptualization, Data curation, Formal analysis, Methodology, Validation, Writing – original draft, Writing – review & editing. JH: Conceptualization, Formal analysis, Investigation, Methodology, Supervision, Validation, Writing – review & editing, Writing – original draft.

## Footnotes

^1^ https://automeris.io/WebPlotDigitizer
