# Supplementary material for: Increased dual-task interference during upper limb movements in stroke exceeding that found in aging – a systematic review and meta-analysis
Source: Front Neurol. 2024 Jul 5;15:1375152. doi: 10.3389/fneur.2024.1375152 (PMC11258041; doi:10.3389/fneur.2024.1375152)
Supplement: Supplementary file 1 [file Table_1.docx]

Supplementary Table 1: Dual-task interference - meta-analysis in stroke: extracted data and task description.

| Study | Dual-task interference:  healthy persons | Dual-task interference:  stroke patients | Tasks description |
| --- | --- | --- | --- |
| Bank et al. (2018) | 4.84 *±* 17.12 | 7.19 *±* 25.62 | P: % of collected targets divided by „catch time“  M: reaching task in frontal plane in VR  C: high complexity  (values calculated from data in supplement, inverse values used) |
| Bui et al. 2019 | NA | 54 *±* 23.67* | P: smoothness of trajectory  M: robot-based tracking task  C: visual digital span, sequence length=4  (z-values used; standard deviation calculated from standard error) |
| Hejazi-Shirmard et al. 2020 | 19.35 *±* 0.008 | 54.1 *±* 0.02 | P: normalized movement time  M: reach and grasp movement  C: difficult auditory backward digit task  (patients with low level of anxiety; Dual-task interference calculated from single- and dual-task values) |
| Houwink et al. 2013 | -0.44 *±* 7.24 | 30.67 *±* 21.67 | P: composite score (area x speed)  M: circular movements in transversal plane, moderate stroke, without arm support  C: auditory Stroop  (values extracted with WebPlotDigitizer; calculated as 100 - extracted values) |
| Kim et al. (2021) | 0.3 *±* 3.4 | 7.8 *±* 9 | P: accuracy  M: circle (o-shaped) movement task with 2-D robot  C: serial sevens (S7)  (inverse values used) |
| Mullik et al. (2021) | 7.4 *±* 21.1 | 28.9 *±* 42.8 | P: success rate  M: reaching task in 3-D VR  C: 1-back task  (inverse values used) |
| Singh et al. (2023) | -0.5 ± 1.3 | 1.8 ± 1.8 | P: mean reach speed  M: trajectory in trail making test  C: Alphanumeric switching |

Values are means±SD of normalized dual-task interference (in %), i.e. relative to single-task performance (see Methods; positive values indicate lower dual-task motor performance). These values are reported in Fig. 2A and respective group differences in the Forest plot. Abbreviations: C: cognitive task; M: motor task; P: parameter quantifying dual-task interference; VR, virtual reality.

Supplementary Table 2: Dual-task interference – meta-analysis in healthy older subjects: extracted data and task description.

| Study | Dual-task interference: healthy young subjects | Dual-task interference: healthy older subjects | Tasks used in meta-analysis |
| --- | --- | --- | --- |
| Kemper et al. (2003) | 17.02 *±* 1.99 | 22.39 *±* 1.53 | P: time-on-task  M: complex 4-finger sequence tapping task  C: talking  (values extracted using WebPlotDigitzer) |
| Petit et al. (2011) | 7.4 *±* 16.3 | 58.0 *±* 50.2 | P: reaction time for left hand  M: uni-manual tapping task  C: letter fluency task  (mean values calculated from individual values) |
| Vaportzis et al. (2014) | 25 *±* 22 | 35 *±* 27 | P: movement speed  M: circle tracing, easy condition  C: serial subtraction, easy condition  (values recalculated; multiplied by 100) |
| Voelcker-Rehage et al. (2006) | 3 *±* 7 | 11 *±* 15 | P: grip force SD  M: precision grip, isometric constant force;  C. visual 2-back task |
| Voelcker-Rehage et al. (2007) | -6.58 *±* 13.38 | 38.43 *±* 33.33 | P: grip force CV (coefficient of variation)  M: precision grip, tracking task  C: auditory 2-back task |

Values are means ± SD of normalized dual-task interference (in %), i.e. relative to single-task performance (see Methods; positive values indicate lower dual-task motor performance). These values are reported in Fig. 2B and respective group differences in the Forest plot. Abbreviations: C: cognitive task; M: motor task; P: parameter quantifying dual-task interference.
